# Suicide prevention starts before the crisis: intervention guidelines for university students

**DOI:** 10.1186/s41155-025-00357-y

**Published:** 2025-07-18

**Authors:** Hareli Fernanda Garcia Cecchin, Sheila Giardini Murta

**Affiliations:** 1 Universidade Federal do Tocantins — UFT, Palmas, TO CEP 77001-090 Brazil; 2 Instituto de Psicologia — IP, Universidade de Brasília — UnB, Brasília, Brazil

**Keywords:** Suicide prevention, Mental health, University students, Higher education, Context and implementation of complex interventions, Intervention guidelines

## Abstract

**Abstract:**

**Background:**

Suicide among university students is a growing public health concern, particularly in low- and middle-income countries. The university setting presents unique challenges and opportunities for implementing effective suicide prevention strategies. Despite the availability of various interventions, these efforts often fail to address the contextual and systemic factors that influence their success.

**Objective:**

Investigate the elements that can support implementing actions to prevent suicide among university students. Using interviews, focus groups, and questionnaires, the study was conducted at a university in the North of Brazil.

**Participants:**

These are 20 undergraduate students, 12 undergraduate course coordinators, 6 technical-administrative staff, and 12 health professionals.

**Method:**

Thematic analysis and the context and implementation of complex interventions (CICI) model were used to analyze the data.

**Results:**

Thematic analysis revealed that political and socioeconomic contexts—such as underfunded mental health services, lack of institutional coordination, and limited financial aid—were critical barriers. Key facilitators included social participation, teacher-student relationships, and actions that promote a welcoming university environment. Implementation concerns included the risk of stigmatization and the need for role clarity among university staff. Stakeholders proposed a range of interventions distributed across ecological, proactive, early, and crisis zones, emphasizing the need for mental health promotion, intersectoral collaboration, and collective program design.

**Conclusions:**

Effective suicide prevention in universities requires a systemic approach that addresses prevention and treatment actions of suicidality. By leveraging the insights of multiple stakeholders and applying context-sensitive frameworks, universities can implement sustainable interventions. This study provides a road map for advancing suicide prevention efforts and illustrates ongoing and comprehensive actions to promote the mental health of university students.

## Introduction

Suicide has become a significant global public health issue, particularly among adolescents and young adults in developing countries (Malta et al., 2021; WHO, 2021). In Brazil, university students face a unique combination of vulnerabilities—including socioeconomic instability, academic pressure, and limited access to mental health services—which increase the risk of suicidal ideation (Júnior et al., 2024; Santos et al., 2017a, 2017b). This scenario highlights the urgency of implementing suicide prevention programs using universal, selective, and indicated strategies (WHO, 2014).

Within the university setting, Drum and Denmark (2012) proposed a framework organizing suicide prevention into three zones—prevention, clinical intervention, and recovery — encompassing a continuum from ecological and proactive prevention to early intervention and crisis management (Table 1). However, most suicide prevention efforts at universities have focused primarily on psychoeducation and gatekeeper programs, which are limited to prevention and early intervention (Cecchin et al., 2022). Other important strategies, such as improving university counseling services (Thompson et al., 2010), restricting access to lethal means (Joffe et al., 2008), or encouraging students to seek help (Rivero et al., 2014), have been underutilized. Recent reviews underscore the importance of broadening the range of available interventions, including ecosystemic and digital approaches, while also emphasizing the crucial role of context in determining their effectiveness (Breet et al., 2021, Cecchin et al., 2022).

**Table 1:** Suicide prevention structure in universities

	**Prevention zone**		**Clinical intervention zone**		**Recovery zone**
Type	Ecological prevention	Proactive prevention	Early intervention	Treatment and crisis intervention	Lapse and relapses intervention
Objective	Changing the physical and social environment to promote mental health	Reduce suicide predisposition in the general population and improve personal resources	Identify cases of an ongoing disorder process and prevent suicidal crises from occurring	Offer treatment for cases of suicidal ideation or crisis	Strengthen recovery and resilience, reducing relapses among students who have attempted suicide

Source: Prepared by the authors based on Drum and Denmark (2012)

**Fig. 1:**
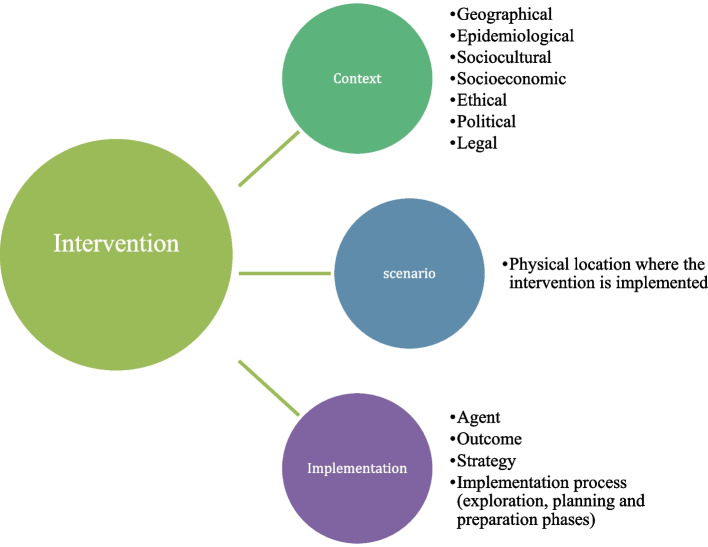
Data analysis process

In this regard, context is not merely the background of interventions—it interacts with implementation and can determine success or failure. The context and implementation of complex interventions (CICI) model offers a systemic perspective (Pfadenhauer et al., 2017). The CICI model includes three interrelated dimensions: context, implementation, and setting. The context dimension encompasses seven domains—geographical, epidemiological, socio-cultural, socio-economic, ethical, legal, and political—that influence how an intervention is perceived and adopted. The implementation dimension includes elements such as implementation strategies, agents (i.e., individuals or organizations involved in putting the intervention into practice), processes, and outcomes. The setting refers to the physical and organizational environment in which the intervention occurs. Therefore, efforts should not be restricted to implementation but should include context analysis, consultation with social actors, and assessment of community resources (Eldredge et al., 2016), to avoid adverse effects (Bonell et al., 2015). Figure 1. CICI model proposed by Pfadenhauer et al. (2017) and was tailored to the university context explored in this study.

The CICI model is viewed as useful in the construction (Zechmeister-Koss et al., 2022) and implementation of programs to prevent negative health outcomes (Abdala et al., 2020). This model emphasizes the importance of engaging local stakeholders to understand contextual facilitators and barriers, which is especially critical in low- and middle-income countries where such factors are often overlooked.

Despite growing awareness, to date, no studies have applied the CICI model to understand suicide prevention in university settings. Most program evaluations overlook how institutional structure, stakeholder engagement, and the broader sociopolitical context influence the reach and sustainability of interventions. The present study addresses this gap by applying the CICI framework to investigate how contextual- and implementation-related factors shape suicide prevention efforts at a Brazilian public university.

Parallel to this methodological innovation, the study also aligns with recent theoretical advances that reframe suicidal behavior not as a purely clinical or intrapsychic issue but as a multifactorial and context-dependent phenomenon. As proposed by Al-Halabí and Fonseca-Pedrero (2024), suicide prevention must incorporate a contextual-functional and existential lens, recognizing the human suffering behind the act. This perspective complements the CICI model (Pfadenhauer et al., 2017) by reinforcing the need for multilevel, multisectoral strategies that address social determinants and humanize care practices.

To respond to these challenges, recent research has highlighted the effectiveness of multifaceted intervention strategies for university students. Digital interventions, in particular, have emerged as valuable tools to improve access to mental health services, especially where financial or logistical constraints limit traditional care (Auerbach et al., 2018; Lattie et al., 2019). When co-designed with users, these platforms are not only more engaging but also more impactful (Oti et al., 2023). Likewise, physical activity-based interventions have shown strong correlations with improved mental well-being and function synergistically with other strategies (Budzynski-Seymour et al., 2020; Donnelly et al., 2024).

Furthermore, specific subgroups within the university population—such as first-generation or high-risk students in engineering and medical programs—require targeted interventions that address their particular stressors (Jensen & Cross, 2021; Morais et al., 2021). This tailoring might include peer mentoring, specialized counseling, and stress management workshops to foster a sense of belonging and improve coping mechanisms (Jensen & Cross, 2021; Morais et al., 2021).

Another promising strategy involves the use of frameworks like Intervention Mapping to design health promotion programs based on stakeholder engagement and context-specific evidence (O’Brien et al., 2020). Enhancing family health literacy has also shown potential in positively influencing students’ mental health (Wang et al., 2023), expanding the scope of intervention beyond campus boundaries.

Given the complexity of these challenges, this study was guided by the following research questions: What contextual elements support or undermine the successful implementation of suicide prevention interventions for university students? What iatrogenic effects can be avoided in the design of these interventions? How can contextual facilitators and barriers inform program development? Which intervention modalities are perceived as appropriate within the local university context?

This study aims to investigate the elements that may support or hinder the implementation of suicide prevention initiatives in a Brazilian university, contributing to the development of context-sensitive and sustainable mental health strategies for higher education institutions. By doing so, it will be possible to identify a set of implementation resources and challenges relevant, thus supporting mental health planners, educators, and decision-makers in designing suicide prevention programs.

## Method

The study followed a sequential exploratory study design, using a mixed-method approach, with multiple informants and a data triangulation strategy (Creswell, 2010), based on the CICI model (Pfadenhauer et al., 2017). The study included four phases of data collection and analysis. Subsequently, the results of both phases were integrated during the interpretation phase. This design was used for a clearer and more comprehensive understanding of elements that can support implementing actions to prevent suicide among university students. The first part, qualitative, interviewed 20 university students. The second part, qualitative, consulted six technical-administrative staff through focus-group sessions. The third part, quantitative, applied a questionnaire to 22 health professionals. The fourth part, qualitative, interviewed 12 course coordinators. The research process used the data analysis from one stage to support the next stage. The study was organized using the mixed methods article reporting standards (MMARS) of the American Psychological Association (APA) (Levitt et al., 2018).

The study, carried out at a public university in the northern region of Brazil, offers undergraduate and postgraduate courses in various areas of knowledge. The university operates in five municipalities, comprising five campuses.

### Participants

The study had 60 participants, including 20 undergraduate students, 6 technical-administrative staff, 12 health professionals, and 12 undergraduate course coordinators. The sample of students included in this study was defined based on a diagnosis of suicidal ideation made by healthcare professionals. Students were identified through the university’s mental health support services, which maintain records of students who seek psychological and psychiatric assistance. Healthcare professionals, including psychologists and physicians, conducted initial assessments of students presenting mental health issues. The diagnosis of suicidal ideation was based on standardized clinical interviews conducted by licensed mental health professionals. These interviews were guided by established diagnostic criteria from the *Diagnostic and Statistical Manual of Mental Disorders (DSM-5)*. Key indicators for diagnosing suicidal ideation included the presence of thoughts about self-harm or suicide, reported plans or means to commit suicide, and any previous suicide attempts. The final sample consisted of students who met the inclusion criteria of being diagnosed with suicidal ideation and belonging to a low-income background. These students had received mental health treatment for 12 months and had documented suicidal ideation. Health professionals were not university staffs; they worked in the private health network and cared for university students described above.

### Instruments

The instruments (interviews, focus group, and questionnaire) were developed based on research questions and on the literature (Pfadenhauer et al., 2017), described in Table 2. Data collection was performed by a researcher with a degree in psychology, 10 years of experience in qualitative research, and familiarity with the institution’s culture.

**Table 2:** Data collection instruments used in each scenario

Public	Instruments	Questions
Undergraduate student	Individual interview	● What do you think could be done at university to improve your mental health?
Technical-administrative staff	Focus group	● What are the opportunities/resources/doors for suicide prevention at university? ● What are the barriers to suicide prevention at university? ● What precautions should be taken when implementing actions to prevent suicide at universities?
Healthcare professional	Questionnaire	● What do you think the university could do to improve student mental health?
Course coordinator	Individual interview	● What could be done to improve university students’ mental health? ● What are the general principles that should be observed when universities offer actions to prevent suicide?

### Recruitment procedures

Students were recruited after consulting the university database. Firstly, data was obtained from undergraduate students who received financial aid from the university to undergo mental health treatment in 2020. One-hundred eighteen students comprised the original sample, 50 of whom had suicidal ideation certified by a private healthcare professional. Twenty students with suicidal ideation were selected. The students with suicidal ideation were selected, focusing on a balanced division between campuses and choosing those who had received financial aid for a longer time. Twenty students were interviewed, 16 women and 4 men. Students were recruited via email and invited for an individual interview via videoconference at the participant’s convenient date and time. The interviews lasted from 17 to 50 min, with an average time of 28 min.

The same database was used to recruit healthcare professionals. There were 41 health professionals who cared for the aforementioned students. These health professionals were recruited via email and received a link to the questionnaire. Twenty-two health professionals (53.6%) responded, and 2 refused to participate citing ethical reasons.

The 37 course coordinators were recruited via email and were invited to participate in an individual interview via videoconference and then asked to choose a convenient date and time. Twelve course coordinators (32.5%) responded.

The six technical-administrative staff were selected based on convenience and on their position in a student assistance center, at the dean’s office of student affairs, the dean’s office of undergraduate studies, or the NZ^1^ program. The technical-administrative staff were recruited via email, requesting one participant per department. Six of the eight invitations were answered.

### Data collection

The study included four data collection phases carried out between May and August 2021. In phase 1, the students who had received financial aid were interviewed. In phase 2, the technical-administrative staff were interviewed in three biweekly focus-group sessions. Phase 3 included an online questionnaire administered to the health professionals who had cared for the students that received the financial aid. The undergraduate course coordinators were interviewed individually in phase 4. These course coordinators were interviewed because of their leadership roles, caring for students and directing them to other sectors. All interviews and focus groups were carried out via videoconference, and the communication was subsequently transcribed speech.

### Data analysis

Data analysis was conducted in three distinct phases, combining both inductive and deductive approaches, as appropriate to the exploratory and implementation-oriented focus of the study. All qualitative data (transcripts of interviews) were analyzed using thematic analysis (Braun & Clarke, 2006). The health professionals’ questionnaire responses were analyzed via descriptive statistics.

In the first phase, two researchers independently conducted initial coding of the data from each target group (students, course coordinators, technical-administrative staffs, and mental health professionals). Through a process of repeated reading and line-by-line coding, meaningful segments of text were identified and assigned preliminary codes. These codes were then discussed and refined collaboratively to ensure consistency and interpretive alignment.

In the second phase, the researchers generated and defined themes inductively based on the coded data. Themes were grouped into five overarching categories that emerged across the dataset: resources, barriers, care, suggestions, and principles for implementing a suicide prevention program at the university. These categories were not predetermined but emerged through inductive analysis of recurring patterns in the participants’ responses. Principles refer to a set of norms and actions that reflect values, overarching guidelines, or broader conceptual frameworks, which should be considered in the design of the intervention ( Tables 3, 4, 5 and 6).

In the third phase, the data were reanalyzed deductively, using two theoretical frameworks. First, the CICI model (Pfadenhauer et al., 2017) was applied to map contextual factors related to implementation. Second, the intervention suggestions were categorized according to the intervention zones proposed by Drum and Denmark (2012) (e.g., universal, selective, indicated interventions), enabling a structured classification of proposals made by different stakeholder groups. A table was created to organize the results to highlight the agreements and divergences between multiple informants (Table 10), following the recommendations of Levitt et al. (2018).

To ensure trustworthiness, we adopted strategies such as triangulation of data sources (students, course coordinators, technical-administrative staff, and mental health professionals) and dual independent coding by researchers. Two researchers independently coded the data, and discrepancies were resolved through consensus meetings, which contributed to both credibility and dependability. Coding decisions were documented through reflective memos, allowing for an audit trail and supporting confirmability.

The member checking procedure, systematized by Birt et al. (2016), was used to evaluate data reliability. A summary was sent to all participants who were asked to read it and comment on whether the results reflected their experiences. In the case of technical-administrative staff, the results were read and commented on in a new focus-group meeting.

*Ethical considerations.*

All participants were fully informed about the objectives of the study, the researchers responsible, the voluntary nature of participation, and their rights, including the right to withdraw at any time without consequences. Informed consent was obtained through written consent forms and an additional authorization form covering the use of images and audio recordings. Participants were also informed about potential risks and benefits, including the possibility of emotional discomfort when discussing sensitive topics. In such cases, emotional support was provided by a qualified professional, and participants were referred to specialized mental health services available at the university, if necessary. Privacy and confidentiality were ensured throughout the research process, especially in the “Results” section, where identifying details were removed or anonymized. The interviews were conducted by a trained researcher who adopted an attentive, sensitive, and supportive approach, fostering a safe environment for open dialogue. The study followed the principles outlined in the Declaration of Helsinki and was approved by the Research Ethics Committee in Human and Social Sciences of the University of Brasília (CAAE nº 42405021.3.0000.5540), in accordance with Resolution nº 510/2016 of the Brazilian National Health Council. No incentives or financial compensation was offered to participants.

## Results

The results are presented according to the dimensions of the CICI model—context, setting, implementation, and intervention. Data was analyzed from four participant groups: undergraduate students (*n* = 20), course coordinators (*n* = 12), health professionals (*n* = 22), and technical-administrative staff (*n* = 6). While the perspectives of technical-administrative staff are particularly prominent in the context and implementation domains due to their institutional roles, the suggestions and insights from students, coordinators, and health professionals are fully integrated into the intervention domain, as shown in Tables 7, 8, 9, 10, 11, 12, 13, and 14.

The undergraduate students were aged between 21 and 32 years old (average of 24.4 years old). The technical-administrative staff members worked at the institution for 3 to 12 years (average of 7 years). The course coordinators had worked at the institution from 0.5 to 17 years (average of 6.6 years). Tables 3, 4, 5, and 6 show the other characteristics.

**Table 3:** Undergraduate students’ sociodemographic and educational data (*n* = 20)

Name	Gender	Sexual orientation	Age	Ethnicity	Course	Course modality
P1	Feminine	Heterosexual	23	Brown	Forest engineering	Full-time
P2	Feminine	Heterosexual	23	Black	Bioprocess engineering	Full-time
P3	Masculine	Heterosexual	24	Black	Letters	Morning
P4	Feminine	Heterosexual	24	Brown	Psychology	Full-time
P5	Masculine	Heterosexual	28	White	Agronomy	Full-time
P6	Feminine	Homosexual	23	Brown	Psychology	Full-time
P7	Masculine	Homosexual	24	Brown	Pedagogy	Morning
P8	Feminine	Heterosexual	27	Black	Social service	Morning
P9	Feminine	Homosexual	23	Black	Journalism	Morning
P10	Feminine	Heterosexual	25	Brown	Environmental engineering	Full-time
P11	Feminine	Homosexual	25	Indigenous	Medicine	Full-time
P12	Feminine	Homosexual	32	Black	Pedagogy	Morning
P13	Feminine	Heterosexual	23	White	Forest engineering	Full-time
P14	Feminine	Heterosexual	21	Brown	Bioprocess engineering	Full-time
P15	Masculine	Heterosexual	21	Brown	Psychology	Full-time
P16	Feminine	Heterosexual	25	Brown	Agronomy	Full-time
P17	Feminine	Bisexual	25	Black	Medicine	Full-time
P18	Feminine	Heterosexual	25	Brown	Psychology	Full-time
P19	Feminine	Homosexual	23	Brown	Psychology	Full-time
P20	Feminine	Homosexual	24	Brown	Social sciences	Morning

**Table 4:** Health professionals’ data (*n* = 22)

Variable	Description	Profession		Total
		**Physician**	**Psychologist**	
Gender	Feminine	12%	87%	100%
	Masculine	66%	33%	100%
Total	––––––––	27%	73%	100%

**Table 5:** Administrative technicians’ sociodemographic and educational data (*n* = 6)

Name	Gender	Education	Location	Time
P1	Feminine	Social service	Dean of Student Assistance	5
P2	Feminine	Psychology	Campus 1	10
P3	Feminine	Psychology	Campus 2	5
P4	Feminine	Social service	Campus 3	3
P5	Feminine	Psychology	Campus 4	12
P6	Feminine	Social service	Campus 5	7

Note: Time is in years and refers to the length of time working at the institution

**Table 6:** Sociodemographic and educational data of course coordinators (*n* = 12)

Nome	Gender	Course	Education	Time
P1	Masculine	Environmental engineering	Doctorate	8
P2	Masculine	Civil engineering	Doctorate	8
P3	Feminine	Law	Doctorate	7
P4	Masculine	Physical education	Master’s	0.5
P5	Feminine	Forest engineering	Doctorate	3
P6	Feminine	Nutrition	Doctorate	8
P7	Feminine	Social service	Doctorate	10
P8	Feminine	Law	Master’s	3
P9	Feminine	Field education	Master’s	4
P10	Feminine	Journalism	Doctorate	17
P11	Feminine	Pedagogy	Doctorate	8
P12	Masculine	Admin/management	Doctorate	3

Note: Time is in years and refers to the length of time working at the institution

### Context dimension

The technical-administrative staff pointed out barriers and facilitators related to the context shown in Table 7.

**Table 7:** Barriers and facilitators in the university context according to technical-administrative staff

Domain	Code	B	F
Geographic	Inadequate physical space to care for students within the university	x	
Sociocultural	Lack of integration between the university and the student’s family to organize medication treatment	x	
Socioeconomic	Students are not covered by financial aid and unable to receive treatment in the private network	x	
	Insufficient financial aid to cover the entire treatment	x	
	Online assistance from the university psychologist as a strategy to increase student trust in the service provider		x
	Psychology course with clinical internship and projects		x
	NZ program		x
Policy	Reduced teams in student assistance centers	x	
	Overworked professionals with no time for continuing education	x	
	Lack of managers’ understanding who overload student assistance staff with activities that are not the responsibility of this sector	x	

Legend: *B* barrier, *F* facilitators

In the geographic domain, the lack of adequate physical space to provide student support is illustrated in the following interview excerpt:

We also face problems related to physical infrastructure — we don’t have an adequate space to serve students, neither in the area of social work nor, even less, in psychology or mental health care. So it’s quite difficult for them to seek us out. And over there, although another public servant and I, both social workers, have a small room, it is very accessible to other staff members. There is no privacy, no isolation — nothing. Anyone nearby can hear what is being said, and other staff members in the common area can see the student coming in for help. I believe this also discourages university students from seeking support (Participant 33, technical-administrative staff).

The context also depicts facilitators in the ethical domain that must be considered when designing suicide prevention programs. Table 8 summarizes the principles used in designing interventions to prevent suicide. The technical-administrative staff mentioned the need to design and implement collective actions to cautiously monitor the construction of an intervention, which was considered a principle.

**Table 8:** Principles for preventing student suicide reported by coordinators

**Category**	**Code**
Promoting health	Develop a culture of respect and empathy among all university actors
	Raise awareness among teachers about the importance of promoting students’ mental health
	Change the physical and social environment so that students perceive the university as a welcoming place
Integrality, transversality, and intersectorality	Perceive the student as an integral human being, understanding that teaching goes beyond technique and that mental health must be addressed in a transversal way
	Establish partnerships between the university and society, as these are complex problems and the university will not solve them alone
Social participation	Develop actions collectively, including teachers in the debate and considering the problems of each course Foster dialogue with students

In the principle of integrality, transversality, and intersectorality, the code “Perceive the student as an integral human being, understanding that teaching goes beyond technique and that mental health must be addressed in a transversal way” is illustrated in the following interview excerpt:

It’s a situation of vulnerability, so sometimes exposing oneself may worsen the situation, leading the student to remain silent. And then we return to what we discussed earlier — how close is the student to the course, to the people who make up the course? Would the student feel comfortable opening up to me… is there space for that? I believe that humanizing the university ends up being a positive element in this whole process. I think the university is not a very humanized environment; we tend to focus more on technical aspects. But the human being is integral — we can’t separate these dimensions. So, when I have a friendly relationship with the student, this happens more naturally. The student becomes close to the professor, even in academic guidance. If there is affinity, there is a greater chance the student will open up (Participant 32 – course coordinator).

### Setting dimension

In the setting dimension, the technical-administrative staff presented a precaution that should be observed when constructing the intervention: security of the service location, avoiding places that could pose a risk if the student has a psychotic crisis or is at risk of suicide. This is illustrated in the following interview excerpt:

Another concern I always have is whether the place where the service is provided ensures safety. For example, there was a time when we were working on the second floor, and I was always very careful to make sure the window was locked to prevent any attempt by someone, during a psychotic episode, to open the window and jump out. When I talk about a suitable location, I’m also referring to this — the safety of the psychologist as well. In this case, since we (psychologists) follow students who have suicidal ideation, persecutory delusions, and access to chemical products, this was something we discussed for a long time. At one point, this student had a psychotic episode on campus and had access to chemical substances — he could have taken acid and thrown it at someone. So we’re also talking about our own safety, and the safety of the campus, because we’re responsible for all of that. Thank God we haven't had any mass attack incidents here, but that’s something I always try to consider: what is available, what kinds of things could pose a threat. At least that’s how I think — when we talk about mental health, it’s not just about the risk a person poses to themselves, but also the risk they may pose to others.

I once provided care to a student many years ago — I was in my first or second year at the university — and this student had suicidal ideation and access to firearms at home. One day, we had to hospitalize him urgently. He showed up on campus armed. The issue was that his ex-girlfriend was now dating someone else on campus. He said to me: “I came to talk to you. I have the gun with me, and if I don’t talk to you, I’m going to do something stupid.” And by “something stupid,” he meant taking the gun, killing them both, and then himself. So this is a constant concern for me, because it seems that this is not obvious to everyone, but mental health is not only about the risk a person poses to themselves — it’s also about the risk they may pose to others, including to us. That’s why I always remain alert to this aspect (Participant 34, technical-administrative staff)

### Implementation dimension

In the implementation dimension, the technical-administrative staffs presented five suggestions related to the implementation process, summarized in Table 9. The exploration phase emphasized the concern of staff members regarding the university not being responsible for services that are the responsibility of the Unified Health System (SUS), such as psychotherapy and intervention in suicidal crises. In the planning phase, interviewees point out the need to be cautious with potential harm to the target audience of the intervention, resulting from the stigmatization, embarrassment, and labeling of university students. In the planning and preparation phase, one technical-administrative staff member emphasized the importance of clearly defining the university’s role in order to avoid actions that might stigmatize students. Her statement illustrates the code “Avoid actions that may cause stigmatization, embarrassment, or labeling of students” as follows:

I also believe it is important, for implementation purposes, to clearly define the university’s role. This role needs to be well established so that mental health and suicide prevention actions are appropriate for this setting — aligned with its goals. I believe this is extremely important because, depending on the type of action, it may lead to increased stigmatization of students, cause embarrassing situations, or result in labeling. In short, I think clear criteria must be established to ensure that any intervention is consistent with the objectives of the academic environment (Participant 37 – technical-administrative staff)

**Table 9:** Care related to the implementation process according to technical-administrative staff

Category	Code
Exploration phase	Stick to the work that belongs to the university
	Consider the available human and financial resources
Planning and preparation phase	Avoid actions that may cause stigmatization, embarrassment, and labeling of students
	Ensure confidentiality throughout the student’s psychotherapeutic process
	Design actions collectively

### Intervention domain

In the intervention domain, the suggestions point to the components and delivery of interventions. All stakeholders who were interviewed provided suggestions for suicide prevention interventions for university students, which are related to implementation strategies. The three intervention suggestions with the highest percentage of responses from students were as follows: Improving the student–teacher relationship (60%), enhancing student assistance (55%), and offering themed groups and discussion circles (30%). The students’ suggestions are detailed in Table 10.

**Table 10:** Undergraduate student suggestions

Code	%
Improve the teacher-student relationship	60%
Improve health assistance	55%
Offer thematic groups and discussion circles	30%
Support students at risk	15%
Create an academic culture focused on health promotion and social connection	15%
Publicize the Student Assistance Center service	10%
Carry out screenings to identify students at risk (e.g., alcohol abuse, depression)	10%
Invest in mental health care actions for teachers	10%
Making changes to the physical space affects students’ quality of life and learning	10%
Reduce discrimination against students through debates on cultural diversity	10%
Welcome incoming students	10%
Offer extension projects that enable coexistence with the community	10%
Partner with services in the region to refer cases	10%
Offer stress management techniques (yoga workshops)	5%
Provide better conditions for students to adapt to remote learning	5%
Avoid academic calendars with very short periods	5%
Carry out mental health campaigns and improve Yellow September	5%
Support students at the end of their studies of course	5%

The three intervention suggestions with the highest percentage of responses from health professionals were as follows: Conducting mental health campaigns (31.8%), offering themed groups and discussion circles (13.6%), and offering psychoeducation programs (13.6%). All suggestions provided by health professionals are presented in Table 11.

**Table 11:** Suggestions from health professionals

Code	%
Conduct mental health campaigns	31.8
Offer themed groups and discussion circles	13.6
Offer psychoeducation programs	13.6
Provide psychotherapy for students in crisis and/or psychological distress	9.1
Offer programs that promote art, culture, and sports	9.1
Monitor students at risk	4.5
Provide stress management techniques	4.5
Conduct screenings to identify at-risk students	4.5
Improve the student–teacher relationship	4.5
Revise curriculum to enhance meaningful learning	4.5
Provide scholarships for students in social vulnerability	4.5
Provide career guidance and planning services	4.5
Modify physical spaces to improve quality of life and student learning	4.5
Reduce discrimination through discussions on cultural diversity	4.5
Offer gatekeeper training programs for professors	4.5
Improve student assistance	4.5
Establish partnerships with local services for case referrals	4.5
Invest in the mental health of university health professionals	4.5

The three intervention suggestions with the highest percentage of responses from course coordinators were as follows: creating an academic culture focused on health promotion and social connection (50%), modifying physical spaces to improve student well-being and learning (41.7%), improving the student–teacher relationship (25%), and conducting mental health campaigns (25%). All suggestions are listed in Table 12.

**Table 12:** Suggestions from course coordinators

Code	%
Create an academic culture focused on health promotion and social connection	50
Modify physical spaces to improve student well-being and learning	42
Improve the student–teacher relationship	25
Conduct mental health campaigns	25
Promote the Student Assistance Center service	17
Improve Student Assistance Center service operations through management support	17
Revise curriculum to enhance meaningful learning	17
Offer programs that promote art, culture, and sports	17
Offer gatekeeper training programs	17
Offer psychoeducation programs	17
Offer themed groups and discussion circles	8.3
Provide psychotherapy for students in crisis and/or psychological distress	8.3
Conduct screenings to identify at-risk students	8.3
Increase research and extension scholarships to retain talented low-income students	8.3
Improve university sector operations for process efficiency	8.3
Create mechanisms to encourage faculty participation in training	8.3
Partner with local health services to offer medical appointments	8.3
Invest in mental health initiatives for professors	8.3
Provide scholarships for students in social vulnerability	8.3
Provide career guidance and planning services	8.3
Create a student leave policy for grieving family loss	8.3

The three intervention suggestions with the highest percentage of responses from technical-administrative staff were as follows: improving the functioning of the Student Support Center through management support (100%), developing guidelines for student mental health programs (60%), improving the student–teacher relationship (60%), and conducting mental health campaigns (60%). All suggestions are listed in Table 13.

**Table 13:** Suggestions from technical-administrative staff

Code	%
Improve the functioning of the Student Support Center through management support	100
Develop guidelines for student mental health programs	60
Improve the student–teacher relationship	60
Conduct mental health campaigns	60
Improve student assistance	40
Improve Student Support Center channels	40
Offer psychoeducation programs	40
Welcome incoming students through senior student mentoring	20
Develop guidelines to restrict access to lethal means	20
Create intervention protocols for crisis situations	20
Promote the Student Support Center service	20
Establish partnerships with family members and significant others for crisis support	20
Partner with local services for case referrals	20
Invest in the mental health of university health professionals	20
Improve tutoring actions to support students with educational gaps	20
Offer academic support to students receiving financial aid	20
Offer themed groups and discussion circles	20
Offer gatekeeper training programs	20
Provide psychotherapy for students in crisis and/or psychological distress	20
Provide career guidance and planning services	20
Offer crisis intervention training for campus directors and course coordinators	20
Conduct screenings to identify at-risk students	20
Restore the NZ program	20
Enable the sharing of student information between the academic office and the Student Support Center	20

Table 14 shows the intervention suggestions in the four audiences that consistently reported the following: improve the teacher-student relationship, offer thematic groups and conversation circles, and carry out mental health campaigns.

**Table 14:** Matrix of suggestions by prevention zone mentioned by participants

Level	Suggestions	S	H	T	C
Ecological prevention	Increase the financial aspect of research and extension scholarships to retain talented low-income students	x	x	x	
	Improve the functioning of student assistance centers (NAE’s) through management support			x	
	Improve the functioning of university sectors to establish agile processes			x	
	Build guidelines for mental health programs aimed at students			x	
	Build guidelines for restricting access to lethal means				x
	Create mechanisms for teachers to participate in training			x	
	Create an academic culture focused on promoting health and social connection	x			x
	Provide more conditions for students to adapt to remote learning				x
	Establish partnerships with health services in the territory to offer medical consultations	x			
	Avoid academic calendars with very short periods	x		x	x
	Invest in actions to care for teachers’ mental health			x	
	Invest in the mental health of university health professionals		x		x
	Improve the teacher-student relationship	x	x	x	x
	Improve monitoring actions to assist students with educational deficits	x	x	x	
	Change the school curriculum for meaningful learning	x			x
	Offer pedagogical support to students receiving student financial aid		x	x	
	Offer scholarships to socially vulnerable students	x	x	x	x
	Offer programs to encourage art, culture, and sports			x	
	Offer career guidance and career planning services		x		x
	Make changes to the physical space for improving student quality of life and learning			x	
	Recompose the NZ program		x		x
	Reduce student discrimination through cultural diversity discussions	x	x	x	x
	Enable transfering student information between the academic secretariat and the NAE's		x	x	x
Proactive prevention	Welcome incoming academics via mentoring from veterans		x		x
	Welcome incoming students		x	x	x
	Monitor end-of-course students	x			
	Offer gatekeeper programs		x	x	x
	Offer psychoeducation programs		x	x	x
	Offer extension projects that enable coexistence with the community	x	x		
	Carry out mental health campaigns			x	
Early intervention	Monitor at-risk students			x	
	Improve NAE’s service channels	x			
	Publicize NAE’s service	x	x		
	Offer themed groups and conversation circles	x			
	Offer psychotherapy to students in crisis and/or mental distress				x
	Offer stress management techniques			x	x
	Conduct screenings to identify at-risk students				x
Crisis treatment and intervention	Improve financial aid for mental health treatment	x	x		x
	Create crisis intervention protocols		x	x	
	Create a leave of absence for students to deal with grief over the loss of family members				x
	Establish partnerships with family and significant others for support during a crisis			x	
	Partner with services in the territory to forward cases	x	x		
	Provide crisis intervention training to campus directors and course coordinators			x	

Legend: *S* students, *H* health professionals, *T* technical-administrative staff, *C* course coordinators

## Discussion

The study investigated which elements, as perceived by university students, course coordinators, technical-administrative staff, and mental health professionals, have the potential to support or undermine a successful intervention implementation to prevent suicide among university students.

The political and ethical domains stand out in the context dimension. These domains are particularly critical in the Brazilian higher education context, where the intersection of limited resources and high demands on student services often results in unmet mental health needs. Addressing these issues requires systemic policy changes and enhanced intersectoral collaboration. The political domain points to university management and the Student Assistance Center teams in terms of service qualification and overloaded teams. This is of concern, given that psychologists tend to be responsible for implementing psychoeducation and gatekeeper programs at universities. The fact that teams are overloaded due to work that is not within their competence demonstrates that the psychologists’ role at the institution may be misunderstood. In a national survey, the following challenges were identified regarding the work of psychologists: insufficient human and financial resources and the managers’ difficulty in understanding the role of psychologists at the university, where 71% of these professionals carry out administrative practices (Almeida et al., 2021). The literature reveals that the work of psychologists in universities is often marked by a lack of clarity regarding the skills and functions expected of these professionals (Moura & Facci, 2016; Santos et al., 2017a, 2017b), a gap that may have started during academic training (Silva et al., 2020). The same issue is highlighted in a study that addresses isolated actions and the lack of an institutional policy, especially regarding ecological prevention (Rodrigues et al., 2020). This lack of documentation highlights a gap in implementation guidance, which the CICI model could address by offering a structured framework to integrate context-specific barriers and facilitators into prevention suicide programs design.

In addition to aligning with existing frameworks, participants also contributed original insights that are not frequently addressed in the literature. For example, technical-administrative staff emphasized the need to ensure physical safety in service locations, especially when attending to students in acute psychological distress. This expands the concept of mental health care infrastructure to include environmental risk management, which is often overlooked in suicide prevention planning.

Furthermore, course coordinators proposed the creation of a student leave policy for those grieving the loss of a family member—a perspective that reflects a compassionate, context-sensitive approach to academic regulation. This suggestion highlights the potential for institutional policies to function as mental health interventions, especially when they address life events that affect students’ emotional well-being and academic engagement.

Another important domain was ethics, since the principles demonstrate a concern related to promoting health, the integrality of human health, the transversality of actions, intersectorality, and social participation. These data are in line with the Okagan Charter of Health Promoting Universities (WHO, 2015) which establishes 10 key principles, including the following: (a) take advantage of strengths, creating opportunities for continuous improvement in health and well-being on campus; (b) use systemic settings an(d approaches, using holistic settings and systems like foci of investigation and intervention; (c) develop transdisciplinary collaborations and intersectoral partnerships, developing collaborations and partnerships within and outside the university; and (d) use participatory approaches, promoting the involvement of students, staff, teachers, administrators, and other decision-makers (WHO, 2015).

The previously mentioned principles must be considered not only as a context but also as the implementation process, guiding the creation of programs, the direction of the university community’s mental health policy, and the change in work processes. The Brazilian Network of Health Promoting Universities began in Brazil recently (Hartmann et al., 2019), which represents an ongoing opportunity to expand and strengthen university involvement in this network, ensuring that its policies and practices are more aligned with these core principles.

Social participation is a principle that shows the involvement of implementing agents, which has concerned scientists in different parts of the world. Several theories guiding the construction of prevention and health promotion programs guide the construction of work groups of researchers and interested parties, ensuring social participation throughout the research process (Eldredge et al., 2016; Wallerstein et al., 2017). The synergy of efforts between researchers and interested parties helps to better use the available resources and strengthen partnerships, maximizing results in the mid and long term, increasing the chances to sustain the intervention over time (Wallerstein et al., 2017). Furthermore, as highlighted in previous research, social support and institutional care are protective factors that align with the principles of intersectionality and social participation emphasized in this study ( Cecchin et al., 2024).

These reflections are consistent with recent theoretical contributions in the field of suicide prevention. Al-Halabí and Fonseca-Pedrero (2024) advocate for a shift away from diagnosis-centered approaches and toward a contextual, phenomenological, and multisectoral understanding of suicidal behavior. According to these authors, suicidal acts should be interpreted as expressions of intense existential suffering, deeply rooted in the person’s biographical, social, and cultural context—not merely as symptoms of mental illness.

This perspective reinforces the idea that universities must go beyond clinical care by promoting institutional practices that humanize services, reduce stigmatization, and incorporate the social determinants of mental health. These include educational, economic, and community-based factors that shape students’ daily experiences. The emphasis on social participation, collective scaffolding, and contextualized care resonates with the suggestions presented in this study and provides further support for designing interventions that are not only evidence based but also meaning-centered and humanizing.

In the implementation dimension, the process domain highlights care related to iatrogenic effects. Bonell et al. (2015) state that similar to clinical interventions, public health programs can also cause harm. According to the authors, in addition to detecting possible damage, it is important to investigate their underlying mechanisms when designing interventions, consulting individuals in the context in which the intervention will be implemented. The goal of the implementation care mentioned by the participants is to avoid what Lorenc and Oliver (2014) classify as psychological (negative psychological impacts) and social (negative social perception of a behavior) damage, ensuring intervention confidentiality and avoiding stigmatization.

The intervention domain emphasizes investing in the teacher-student relationship and offering collective interventions, highlighted by all participants. And in the teacher-student relationship, the literature shows that social support favors fitting in (Wenjing Li et al., 2020) and the student’s permanence in higher education (Dorrance Hall et al., 2017; Hagenauer & Volet, 2014; Oliveira et al., 2014). The teacher’s educational social skills, a pillar that supports the quality of teacher-student relationship, is important for the young person’s mental health, as it is a source of social support for the university student (Vieira-Santos et al., 2018). This social support is vital for students from lower-income families who face academic delays when entering university, as well as social difficulties in remaining at the institution (Almeida et al., 2012; Vieira-Santos et al., 2019). Thus, workshops could offer developing educational social skills for university teachers, focusing on improving the teacher-student relationship.

On the other hand, mental health campaigns were included in the participants’ suggestions. Some students suggested expanding coverage of the Yellow September campaign, as well as reviewing some strategies to make it more attractive to young people. In Brazil, the Yellow September campaign has been the target of disagreements. However, it appears that the campaign did not significantly impact young people (Oliveira et al., 2020), stressing that the approach was ineffective to the topic, especially on social media (Lima & Brandão, 2021). Therefore, it is crucial to develop such initiatives, considering specialized guidelines (Ministério da Saúde, 2018; WHO, 2021).

The scope of the intervention brought a distance between conception and practice. A cross-sectional view of the information suggested by technical-administrative staff demonstrates that the responses are concentrated in the clinical intervention area. In terms of context, setting, and implementation, the suggested information demonstrates a concept of suicide prevention focused on individual psychotherapy. Although health professionals and managers have discussed prevention strategies for students since 2005 (Tavares et al., 2008), almost two decades later, it was observed that these ideas were not implemented in the national context. Psychotherapy and counseling are some of the main activities carried out by IFES psychologists, albeit they recognize the importance of prevention and actions to promote health (Almeida et al., 2021).

These results show a paradox in the sense that while staffs highlight that the university should not offer health treatment services, the resources and barriers mentioned are related to the clinical intervention zone. It is possible that the distance between conception and practice may be reinforced by institutional elements. For technical-administrative staffes, it may be more viable to work with individual assistance than to address issues linked to institutional functioning, such as: academic calendar, course curricular structure, teacher-student relationship, and others. The literature points to organizational barriers that hinder organizing prevention actions at the ecological level (Almeida et al., 2021; Rodrigues et al., 2020), and future studies can delve deeper into this issue.

In any case, efforts to implement a suicide prevention program must actively strive to clarify the roles of the tasks and responsibilities of the different professionals on the student assistance team, especially psychologists. Role clarity and transparency of responsibilities can be an important step for the sustainability of the program at the institution and for dissemination efforts. There is emphasis on the need to encourage training professionals so they can recognize the different levels of intervention, especially the prevention zones prior to illness (ecological and proactive prevention), in addition to creating spaces for exchanging information and experiences.

Breaking away from the clinical paradigm—where the focus is exclusively on treatment and individualized interventions—can bring significant benefits to the academic community. The exclusive use of the crisis intervention model means that the task of suicide prevention falls exclusively within the scope of the university’s mental health services, which are in the student assistance centers (Drum & Denmark, 2012). Or at most, it spreads to psychotherapy and whoever can provide it, whether at the university (through the assistance of the dean of student affairs) or in the territory (through the public health system). When crisis intervention is prioritized and prevention is neglected, there is a higher investment of resources, and the team may experience overload with an overflow of urgent and complex demands, without addressing the causes of the problem. Furthermore, palliative interventions in times of crisis do not contribute to constructing a healthy academic environment (Drum & Denmark, 2012; Rodrigues et al., 2020).

Therefore, most of the data collected in the intervention domain focuses on ecological and proactive prevention. It is noted that the suggestions go beyond classic suicide prevention actions, offering the university a range of actions. They can be used as support to build a mental health policy, aimed not only at students but also at teachers and administrative technicians. Some universities have successful experiences in this aspect, such as offering integrative community therapy remotely (Polejack et al., 2021) and the subject on happiness with mental health themes addressed in the classroom (Bernardes & Rosa, 2020). These initiatives offer a welcoming space and mental health literacy, strengthening friendships among students.

These initiatives can encompass the university’s relations with society. Considering that the university is responsible for educating new health professionals, this discussion is encouraged between the sectors responsible for teaching, so as to change the curricular structure of health courses. This could cover themes related to prevention, educating professionals to skillfully work in public health programs. This is especially relevant because a literature review showed that 60% of publications on suicide prevention in primary health care misuse the term prevention, which is linked to other areas of professional activity (Gotti et al., 2022). Another literature review on the state of the art of suicide prevention in Brazil points to descriptive and correlational studies, specific mental health actions, and the lack of systematic evaluation of interventions carried out to prevent suicide, making it difficult to disseminate good practices (Rodrigues et al., 2020).

This study was carried out with a small and context-specific sample (a single university in northern Brazil). While useful for exploratory insights, this limits the generalizability of the findings. Another limitation is that there were no interviews with managers. For future studies, the suggestion is to replicate research in several institutions in order to identify the needs of universities in the same geographic region and develop guidelines for student mental health policy. The context can be raised through a focus group with different professionals, such as intermediate managers from the dean’s office of student affairs and members of the student assistance policy. The social support received from teachers may also be examined. Finally, consider financing and strengthening universal coverage of the public health system to expand access to mental health services. These efforts can inform the creation of intersectoral policies tailored to the regional context and promote institutional responsibility for student well-being. Strengthening mental health infrastructure within universities may also contribute to early identification and support for students at risk.

The construction of preventive and health promotion actions in universities is defended both internationally and nationally (Universidade de Brasília, 2018; WHO, 2015). This concern has been reinforced by the Health Promoting Universities movement, with key actions directed at supporting personal development, promoting health through research, teaching, and training actions (WHO, 2015).

## Conclusions

The present study spearheaded investigating elements for building a suicide prevention program for university students. It was innovatively based on the CICI model, which has the advantage of going beyond intervention components, considering how contextual resources and barriers can undermine or enhance new interventions, allowing us to elucidate the research questions of this study.

The research results point to two propositions for practice: presenting the results to university managers and building a working group to implement the suggestions presented. The findings offer a guide for continuous and comprehensive mental health actions. It allows going beyond the initiatives most frequently applied in Brazilian universities, which are mental health campaigns such as Yellow September—and providing treatment for students at risk of suicide. This study aligns with findings that emphasize the role of institutional risk factors for suicidal ideation among university students, including academic overload, professor-student relationship conflicts, failed institutional welcoming, and bullying at university which require targeted interventions for ecological prevention strategies (Cecchin et al., 2024). However, the literature reveals that efforts to address this issue are often hampered by the absence of robust epidemiological data and insufficient consideration of the cultural context surrounding student suicides (Khan et al., 2024). These barriers highlight the need for comprehensive surveillance systems and sustainable, culturally sensitive interventions.

Prevention efforts are more likely to fail when applied only during crises, as they miss the opportunity to reach students before the onset of suicidal ideation. Suicide prevention strategies should promote student well-being and resilience, ensuring a broader and lasting impact. The successful implementation of these strategies depends on three pillars: (a) Coordinated institutional engagement across multiple levels, (b) the inclusion of diverse stakeholders, and (c) continuity of care integrated into institutional policies. Universities must invest in training for staff and faculty, strengthen partnerships with public services, and form intersectoral coalitions that embed mental health into the university’s mission.

These findings reinforce the need for higher education institutions to move beyond crisis-centered approaches and adopt comprehensive, context-sensitive strategies for suicide prevention. The results support the development of institutional mental health policies that integrate ecological and proactive actions, such as improving faculty-student relationships, enhancing service coordination, and promoting a culture of well-being. They also point to the importance of forming intersectoral working groups to guide program implementation. These efforts can strengthen prevention capacity, particularly in low- and middle-income countries, and align university practices with broader public health policies.

## Data Availability

Datasets used and/or analyzed during the current study are available from the corresponding author upon reasonable request.

## Abbreviations

APA: American Psychological Association
CICI: Context and implementation of complex interventions
DSM-5: *Diagnostic and Statistical Manual of Mental Disorders*
MMARS: Mixed methods article reporting standards
SUS: Sistema Único de Saúde (Brazilian Public Health System)

## Footnotes

1 Suicide prevention program whose name was changed to avoid identification of the university.
